# Oncologic and Functional Outcomes After Primary and Salvage Laryngopharyngoesophagectomy With Gastric Pull-Up Reconstruction for Locally Advanced Hypopharyngeal Squamous Cell Carcinoma

**DOI:** 10.3389/fonc.2019.00735

**Published:** 2019-08-06

**Authors:** Jeroen Meulemans, Floor Couvreur, Eline Beckers, Philippe Nafteux, Hans Van Veer, Vincent Vander Poorten, Pierre Delaere, Willy Coosemans

**Affiliations:** ^1^Otorhinolaryngology-Head and Neck Surgery, University Hospital Leuven, Leuven, Belgium; ^2^Section Head and Neck Oncology, Department of Oncology, KU Leuven, Leuven, Belgium; ^3^Thoracic Surgery, University Hospital Leuven, Leuven, Belgium

**Keywords:** hypopharynx, squamous cell carcinoma, laryngopharyngoesophagectomy, gastric pull-up, salvage surgery

## Abstract

**Background/Purpose:** Hypopharyngeal squamous cell carcinomas (SCC) are generally diagnosed in an advanced disease stage. A total laryngopharyngoesophagectomy with gastric pull-up reconstruction is a time tested surgical treatment in our centre for resectable failures or recurrences after primary treatment with organ preservation protocols (radiotherapy or chemoradiation), or as a primary surgical treatment for very advanced hypopharyngeal tumors. We present the results of our approach in terms of success rate, postoperative complications and functional and oncologic outcomes.

**Methods:** We retrospectively reviewed the charts of all patients with hypopharyngeal SCC, who underwent laryngopharyngoesophagectomy with gastric pull-up reconstruction during the period 1989–2015.

**Results:** The cohort included 60 patients. Mean follow-up was 32 months. Stage III and stage IV disease was present in 35 and 60% of patients, respectively. Successful reconstruction by intended gastric transposition was possible in 98.3% of cases. The in-hospital mortality rate was 8.3%. Two-year and five-year actuarial overall survival were 39.5 and 21.1%, respectively. Two-year and five-year actuarial disease specific survival were 58.5 and 46.6%, respectively. Two-year and five-year actuarial locoregional recurrence free survival were both 49.5%. A significantly lower locoregional recurrence free survival was observed in patients with pN+ disease compared to pN0 (Log rank, *p* <0.05). Complete oral intake was achieved in 82.7% of patients. Speech rehabilitation by means of Provox® puncture or electrolarynx was achieved in 66% of patients.

**Discussion/Conclusion:** Total laryngopharyngoesophagectomy with gastric pull-up reconstruction for advanced stage hypopharyngeal SCC combines relatively good oncologic and functional outcomes in a prognostically unfavorable patient group.

## Introduction

Hypopharyngeal squamous cell carcinoma (SCC) accounts for ~3–5% of all head and neck squamous cell carcinomas (1, 2). Patients usually present with advanced stage disease, with stages III and IV in ~17–27% and 57–68.5% respectively, according to some large series (2, 3). High rates of regional metastases are observed: ~60–80% of patients have clinically metastatic cervical lymph nodes at the time of diagnosis, with contralateral occult nodal metastases present in 40% of cases presenting with clinical involvement of the ipsilateral neck (4). Furthermore, systemic metastasis, either diagnosed at presentation or during follow-up, is observed in up to 60% of the patients (5). Due to these high rates of regional and distant disease, hypopharyngeal cancer has the worst survival rate of all head and neck cancers, with reported 5-year overall survival rates between 15 and 45% (1, 6). Traditionally, the treatment of choice for operable hypopharyngeal SCC consisted of radical open surgery (total laryngopharyngectomy or total laryngectomy with partial pharyngectomy) followed by adjuvant irradiation depending on pathologic risk factors. After publication of promising results concerning oncologic outcome and laryngeal preservation in patients with advanced laryngeal cancer treated with induction chemotherapy plus radiotherapy and later concurrent chemoradiation, indications for these non-surgical larynx sparing treatments were expanded to hypopharyngeal tumors (7, 8). As a result, primary treatment of hypopharyngeal SCC gradually shifted from radical surgery toward non-surgical treatment. However, in cases of extensive disease with already compromised laryngeal and hypopharyngeal function, chemoradiation is likely to cause organ preservation without preservation of function. In contrast, radical surgery with adequate reconstruction and adjuvant radiotherapy may lead to improved oncologic and functional outcomes in these highly selected patients (6). Additionally, surgery remains the preferred therapeutic option as a salvage treatment for local or locoregional failures after initial chemoradiotherapy (9).

Both indications frequently necessitate a total laryngopharyngectomy, resulting in a circumferential hypopharyngeal defect. Various reconstructive options are at the head and neck surgeon's disposal, e.g., tubulated (myo)cutaneous free flaps (anterolateral thigh (ALT) flap, radial forearm (RF) flap), free jejunal flap, and gastric transposition or gastric pull-up procedure (9). The aim of this retrospective series is to review oncologic and functional results after total laryngopharyngoesophagectomy with gastric pull-up reconstruction for treatment of locally advanced hypopharygeal SCC, both in the primary and salvage setting.

## Patients and Methods

### Patients

A retrospective study was conducted at an academic tertiary referral hospital (University Hospitals Leuven, Leuven, Belgium). This study was approved by and carried out in accordance with the recommendations of the Institutional Review Board (University Hospital Leuven Committee for Medical Ethics). Informed consent was waived given the retrospective nature of this study. The records of all patients who underwent a total laryngopharyngoesophagectomy with gastric transposition (gastric pull-up or GPU) between 1980 and 2016 were retrospectively reviewed and analyzed (*n* = 211). As they did not fit the purpose of this study, patients fitting the following criteria were excluded from further analysis: patients with tumor recurrences in the neopharynx after total laryngectomy (*n* = 9); tumors with primary site other than the hypopharynx (larynx (*n* = 7), cervical esophagus [*n* = 50) or the thyroid gland (*n* = 3)]; non-squamous cell carcinoma of the hypopharynx (*n* = 1); gastric pull up surgery performed prior to 1989 (*n* = 45) since no sufficient follow-up data were available for this subgroup; gastric pull-up surgery for a non-oncological reason, e.g., benign stenosis of the neopharynx after total laryngectomy and radiotherapy (*n* = 16). Eventually, a total of 60 patients who underwent a total laryngopharyngoesophagectomy with gastric pull-up for SCC of the hypopharynx between 1989 and 2015, were withheld for analysis.

### Treatment

Primary setting laryngopharyngoesophagectomy is defined as upfront surgery performed for a previously untreated hypopharyngeal carcinoma, while salvage setting laryngopharyngoesophagectomy is considered surgery for persistent or recurrent disease after primary (chemo)radiotherapy or it is considered surgery for a second primary SCC in the hypopharynx after previous head and neck (chemo)radiotherapy for a non-hypopharyngeal malignancy with inclusion of the hypopharyngeal area in the irradiation field.

The decision to submit a patient with hypopharyngeal SCC to total laryngopharyngoesophagectomy with gastric pull-up always resulted from discussion during a multidisciplinary tumor board meeting with presence of head and neck surgeons as well as thoracic surgeons, medical oncologists, radiation oncologists, and radiologists. In our center, the main indication to perform a total laryngopharyngoesophagectomy with gastric pull-up is advanced hypopharyngeal SCC extending in the cervical esophagus below the level of the inferior part of the cricopharyngeal muscle (in the primary setting). In salvage cases, due to the well-known problems of submucosal tumor spread and skip lesion in irradiated tissue, we tend to be more aggressive and add an esophagectomy to the circular laryngopharyngectomy whenever the hypopharyngeal SCC is invading the cricopharyngeal muscle. Another indication is any advanced hypopharyngeal SCC necessitating circular laryngopharyngectomy, combined with a second primary carcinoma of the esophagus. For advanced hypopharyngeal SCC not meeting these criteria, our surgical treatment of choice is circular laryngopharyngectomy with free jejunal transfer. Prior to surgery, patients were properly staged and screened for distant disease, including CT or MRI of the neck, whole body PET-CT or CT of the thorax-abdomen (ultrasound of the abdomen and plain chest radiograph in the early patients), flexible esophagogastroduodenoscopy or panendoscopy under general anesthesia. Tumor stage was determined according to the Union for International Cancer Control (UICC) staging system for malignant head and neck tumors, the edition being the one relevant at the time of diagnosis. In our centre, the surgery is performed in a joint collaborative effort between a head and neck surgery and a thoracic surgery team. Tumor resection is performed by the head and neck team and basically involves a total laryngopharyngectomy with bilateral clearance of lymph node station levels VI and VII. In cN0 cases, bilateral elective dissections of levels II-III-IV are usually performed. In the N+ neck, an ipsilateral modified radical neck dissection (MRND) type I or II and contralateral elective dissection is the preferred option. A hemi- or total thyroidectomy is performed in cases with subglottic extension or invasion through the laryngeal cartilage framework. After mobilization of the tumor block, the resected laryngopharynx remains in continuity with the esophagus, which is maximally freed from the membranous part of the trachea through the bilateral cervicotomy. Via a midline or bi-subcostal laparotomy, the thoracic surgery team mobilizes the stomach with preservation of the right gastric and right gastro-epiploic vessels. To maximize the extension of the stomach, usually a duodenal mobilization (Kocher's maneuver) is performed. The gastro-esophageal junction is divided with a trilinear stapler and oversewn with a non-resorbable suture. An umbilical thread is attached to the lower edge of the distal esophagus, after which the specimen can be removed and the thread indicates the retromediastinal route. Subsequently, the stomach is transposed from the abdomen through the posterior mediastinum into the neck, were a pharyngogastric anastomosis is performed between the oropharynx and the gastric fundus (and not onto the gastro-esophageal junction), again in order to achieve sufficient bridging to perform a tension-free anastomosis. In patients with a history of prior head and neck radiotherapy, a pectoralis major muscle flap is used as an onlay flap to cover the pharyngogastric anastomosis, in an attempt to promote healing and avoid pharyngocutaneous fistula (PCF) formation. A jejunostomy is left *in situ* for nutritional support during the postoperative phase. Postoperatively, patients are transferred to the post-anesthesia care unit or intensive care unit, until their medical condition is favorable enough for transfer to the surgical ward. A nil per os policy is maintained until subsequent upper gastrointestinal tract radiographs with low osmolar iodine contrast (Gastrografin®) (postoperative day 5) and with barium sulfate at postoperative day 7 show favorable healing without anastomotic dehiscence or PCF formation, whereupon patients gradually start oral intake. The first postoperative upper gastrointestinal tract radiograph series at day 5 is performed in order to exclude major anastomotic dehiscence. As diagnosis of a large anastomotic defect with barium sulfate could provoke mediastinitis or peritonitis in case the contrast medium escapes from the gastrointestinal lumen and follows the transposed stomach up to the mediastinum/peritoneum, Gastrografin® is the contrast medium of choice for the initial exam of the upper gastrointestinal tract integrity. However, although Gastrografin® has fewer side effects while diagnosing a major leak, this contrast medium is lighter and penetrates less as compared to barium; barium having superior physical properties of mucosal coating and radiographic density (10). Therefore, very small defects might be overlooked, for which barium finds its place in the diagnostic routine following surgery at day 7. Speech rehabilitation starts after hospitalization with electrolarynx speech (Servox®) and professional assistance of a dedicated speech and language pathologist. In primary cases, adjuvant therapy (radiotherapy or combined chemo-radiation) is administered based on the definitive pathological assessment of the resected specimen. The decision to submit the patient to adjuvant therapy always results from a multidisciplinary oncological board discussion. Postoperatively, clinical follow-up is organized at 2-month intervals during the first 2 years, at 3-month intervals during the third year, at 4-month intervals during the fourth year and at 5-month intervals during the fifth year. Baseline imaging (usually CT of the neck) is performed 4 months postoperatively and is repeated 1 and 2 years after treatment. Chest imaging (plain chest radiograph and for more recent patients CT chest) is performed annually to exclude metachronous lung malignancies or distant disease. If indicated, a PET-CT scan is ordered. Concerning speech revalidation, secondary placement of a trachea-gastric speech prosthesis (Provox®) is considered during follow-up on an individual basis.

### Data and Statistical Analysis

The data related to patient, tumor, and treatment characteristics and oncologic and functional outcomes were retrieved from the patient's health records and anonymously stored in an electronic database. Data were collected on gender, ethyl and smoking history, previous treatment for head/neck malignancies, previous treatment for hypopharyngeal malignancies, cTNM classification, operative success rate, tumor histology, pTNM classification, duration of tube feeding (via jejunostomy) and delay to oral intake, hospitalization duration, complications, adjuvant treatment, achievement of speech rehabilitation and technique of postoperative speech, length of follow up, tumor recurrence (local, regional, locoregional, and distant) and cause of death (disease related vs. non-disease related). Success rate was defined as the proportion of patients in whom the gastric transposition resulted in achievement of reconstruction of a patent upper digestive tract, which is related to a preserved sufficient blood supply to the proximal end of the transposed stomach. Postoperative complications were collected and attributed to two categories: early complications (in-hospital complications) and late complications (complications after discharge). The early complications were subsequently divided into the ones who occurred in the head and neck region, and the complications related to the thoracic and abdominal region. Swallowing rehabilitation was considered successful when the patient achieved complete caloric oral intake and as such was independent of tube feedings. We considered speech rehabilitation as successful, once the patient was able to achieve functional communication by speech. We did not objectively evaluate voice quality.

Data were statistically analyzed using SPSS version 22.0 statistical software (IBM corp, Armonk, NY, USA*)*. Complication rates and functional outcomes between different subgroups were compared using 2-sided Fischer's exact test. Kaplan-Meier methods were used to estimate actuarial overall survival (OS), disease-specific survival (DSS), disease-free survival (DFS), locoregional recurrence-free survival (LRRFS) and distant disease-free survival (DDFS). Univariate analysis using log-rank testing was performed to evaluate the association of these outcomes with the levels of different potentially prognostic factors. Statistical significance was defined at the *p* < 0.05 level. Because of the relatively small study population and small subgroups (salvage vs. primary surgery group), multivariate analysis was not possible.

## Results

### Patient Characteristics

The population of 60 patients consisted of 49 males (81.7%) and 11 females (18.3%). Mean age at the time of surgery was 60 years (range 40–79 years, SD = 9.0 years, interquartile range 13 years). Of the 55 patients with known smoking status, 50 (90.9%) were active or former smokers, while 5 patients (9.1%) had never smoked. Concerning the general physical status of the patients before surgery, ASA (American Society of Anesthesiologists) score distribution was score 1 in 13.3%, score 2 in 45%, score 3 in 31.7% and score 4 in 1.7% of patients. ASA score was unknown in 8.3% of patients. Eight patients (13.3%) were tracheotomy-dependent before surgery.

### Tumor Characteristics

Detailed data on cTNM and pTNM classification as well as on staging groups for both the up-front and salvage groups are depicted in Table 1. Although all patients were properly staged preoperatively and considered free of distant metastases at the time of surgery, 1 patient was staged postoperatively with stage IVc disease due to postoperative discovery of distant metastases (M1).

**Table 1:** Overview of tumor characteristics for both the primary and salvage populations.

**Characteristic**	**Primary Group**	**Salvage Group**
	***N* (%)**	***N* (%)**
**Clinical tumor classification**	37	23
*cT2*	1 (2.7)	3 (13.0)
*cT3*	14 (37.8)	15 (65.2)
*cT4a*	21 (56.8)	5 (21.7)
*cT4b*	1 (2.7)	0
**Clinical nodal classification**	37	23
*cN0*	14 (37.8)	10 (43.4)
*cN1*	8 (21.6)	7 (30.4)
*cN2a*	1 (2.7)	0
*cN2b*	6 (16.2)	4 (17.4)
*cN2c*	7 (18.9)	2 (8.7)
*Missing data*	1 (2.7)	0
**Clinical tumor stage**	37	23
*II*	1 (2.7)	2 (8.7)
*III*	10 (27.0)	11 (47.8)
*IVa*	25 (67.6)	10 (43.5)
*IVb*	1 (2.7)	0
**Pathological tumor classification**	37	23
*pT1*	0	1 (4.3)
*pT2*	1 (2.7)	3 (13.0)
*pT3*	10 (27.0)	6 (26.1)
*pT4a*	26 (70.3)	12 (52.2)
*pT4b*	0	1 (4.3)
**Pathological nodal classification**	37	23
*pN0*	11 (29.7)	14 (60.9)
*pN1*	6 (16.2)	3 (13.0)
*pN2a*	1 (2.7)	0
*pN2b*	11 (29.7)	5 (21.7)
*pN2c*	8 (21.6)	1 (4.3)
**Pathological tumor stage**	37	23
*I*	1 (2.7)	1 (4.3)
*II*	1 (2.7)	2 (8.7)
*III*	3 (8.1)	5 (21.7)
*IVa*	31 (83.8)	14 (60.9)
*IVb*	0	1 (4.3)
*IVc*	1 (2.7)	0

### Treatment Characteristics and Complications

Thirty-seven patients (61.7%) underwent laryngo-pharyngoesophagectomy with gastric transposition as an up-front treatment while 23 patients (38.3%) were treated in a salvage setting for recurrent or second primary hypopharyngeal cancer. Surgery for salvage treatment of a second primary hypopharyngeal SCC after the patient had been irradiated previously for another head and neck cancer was performed in 13 cases (21.7%), while 10 patients (16.7%) underwent surgery for salvage treatment of a local recurrence after primary radiotherapy (*n* = 8) or chemoradiation (*n* = 2). One upfront-treated patient (1.7%) received neo-adjuvant chemotherapy prior to surgery. An onlay pectoralis major muscle flap was used to cover the pharyngogastric anastomosis in 10 cases (16.7%). Postoperative in-hospital mortality rate was 8.3% (*n* = 5). Mean life span of these unfortunate patients was 52.6 days (range 25–77 days, SD = 22.6 days). Causes of death were: multi-organ failure (*n* = 1), sepsis (*n* = 1), tracheal sputum impaction with resulting respiratory failure and cardiac arrest (*n* = 1) and sudden cardiac death (*n* = 1). For 1 patient, the cause of death could not be retrieved. After excluding these patients, mean hospitalization duration was 27.6 days (range 11–91 days, *SD* = 17.6 days). In the overall study-population, 95.0% (*n* = 57) of patients were transferred to the intensive care unit (ICU) postoperatively with a mean duration of stay of 5.7 days (range 1–77 days, *SD* = 14.0 days). After discharge from the ICU, 10 patients of these 57 (17.5%) needed to be readmitted to the ICU. Success rate of the reconstruction by gastric transposition was 98.3% with failure of the pharyngogastric anastomosis due to compromised vascularization of the proximal part of the transposed stomach in 1 patient. This patient was salvaged by a free jejunal transfer between the pharynx and the remaining proximal part of the transposed stomach with preserved adequate vascularization. In total, 9 early head and neck complications were recorded in 9 different patients (15.0% of population), necessitating 3 early (in-hospital) re-interventions (5%). Twenty-one patients (35% of total population) experienced a total of 23 early thoraco-abdominal complications. These complications necessitated 11 early re-interventions. Seven late complications related to the head and neck region were recorded in 5 patients (8.3% of the total population), necessitating 6 surgical reinterventions. Table 2 gives a detailed overview of type and incidence of early (in-hospital) and late postoperative complications and related reinterventions in the head and neck region as well as in the thoraco-abdominal region. Adjuvant treatment was administered in 54% (*n* = 20) of primary treated patients and consisted of adjuvant irradiation (51.4%, *n* = 19) or adjuvant concurrent chemoradiation (2.7%, *n* = 1). In the salvage population, 13.0% (*n* = 3) of patients were re-irradiated after surgery.

**Table 2:** Table giving an overview of type and incidence of early (in-hospital) and late postoperative complications and related reinterventions in the head and neck region as well as in the thoraco-abdominal region.

	**Complication incidence (*n*)**	**Reintervention incidence (*n*)**
Early HN complications	- Failure of pharyngogastric anastomosis (1) - Pharyngocutaneous fistula (5) - Postoperative infection with abcedation (2) - Postoperative infection without abscedation (1)	- Free jejunal transfer (1) - Abscess drainage (2)
Early TA complications	- Postoperative pneumonia (8) - Wound-infection (3) - Laparotomy wound dehiscence (5) - Pleural effusion (7)	- Abscess drainage (2) - Wound revision for dehiscence (3) - Intrathoracic drain placement (6)
Late HN complications	- Severe stenosis of the pharyngogastric anastomosis (1) - Tracheostomy stricture (4) - Persistent pharyngocutaneous fistula (2)	- Free jejunal transfer for stenosis (1) - Tracheostomy revision (3) - Fistula resection (2)

*HN, head and neck; TA, thoraco-abdominal*.

### Functional Outcomes

Data on postoperative swallowing function were available for 52 patients (86.7% of population). Complete oral intake was achieved in 43 patients (82.7%) while 9 patients (17.3%) remained completely or partially dependent on their jejunostomy-tube. Continuing feeding tube dependence was higher in the salvage group when compared with the primary group (30.0 vs. 9.4% respectively), but this difference did not reach statistical significance on Fisher's exact test (*p* = 0.071). Data on postoperative voice rehabilitation were available for 50 patients (83.3% of the population). Overall success rate of speech rehabilitation was 66.0% (*n* = 33) and was achieved by electrolarynx (Servox®) in 16 patients (48.5%) and by “tracheogastric” puncture with indwelling voice prosthesis in 17 patients (51.5%). Secondary tracheagastric puncture with introduction of a voice prosthesis (Provox®) was performed in 25 patients (15 primary patients and 10 salvage patients) after a mean postoperative time interval of 5.7 months (range 2–14 months, SD = 3.5 months). Of these patients with a voice prosthesis, 17 (68.0%) achieved a functional speech rehabilitation. This success rate was not significantly different between primary and salvage cases (73.3 vs. 60.0% respectively, *p* = 0.667 on Fishers's Exact Test). However, during follow-up, voice prosthesis removal proved necessary in 6 patients (24% of the “voice prosthesis” group) due to prosthesis related problems such as continuing peripheral leakage combined with the fact that in 5 of these patients, speech was not achieved with the prosthesis. Mean time interval between tracheogastric puncture and prosthesis removal was 14 months.

### Oncological Outcome and Survival

Mean and median follow-up length for the total population (excluding in hospital deaths, *n* = 5) was 32 and 18 months, respectively (range 1–272 months, SD = 47 months). Death occurred in 45 patients (75.0% of total population). In the up-front surgery group (*n* = 37), death occurred in 30 patients during follow-up: 4 deaths resulted from early or in-hospital treatment-related complications. Local or locoregional recurrence and/or distant disease were responsible for 16 deaths, and in 10 patients, death was considered non-disease and non-treatment related. In the salvage surgery group (*n* = 23), 15 deaths were encountered during follow-up, with one in-hospital death, 6 disease-related deaths and 8 deaths which were considered neither non-disease nor treatment related. Concerning disease control in the up-front group (*n* = 37), 5 patients developed locoregional recurrence, 5 patients were affected by distant disease and 11 were diagnosed with synchronous locoregional recurrence and distant metastases. In the salvage group (*n* = 23), 6 patients developed locoregional recurrence, 2 were affected by distant metastases and 2 with combination of locoregional recurrence and distant disease. This spectrum of disease recurrence did not show statistical significant differences between both groups (Chi-square *p* = 0.120).

**Figure 1:**
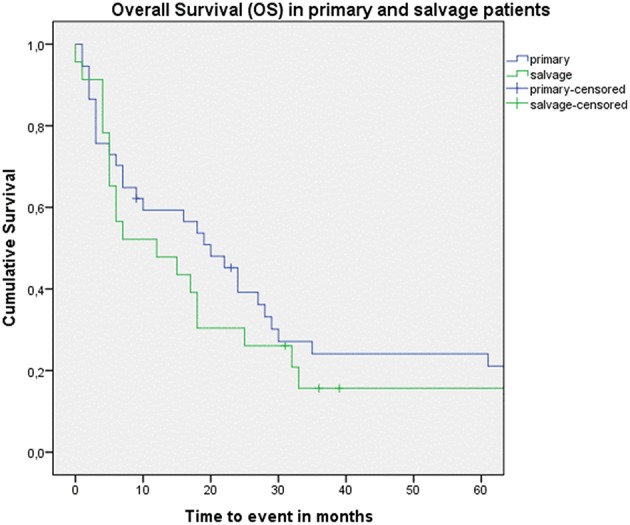
Kaplan–Meier curve illustrating overall survival (OS) in patients treated with up-front or primary surgery (blue) and salvage surgery (green). No difference in OS between both groups is observed (log-rank test, *p* = 0.592).

**Figure 2:**
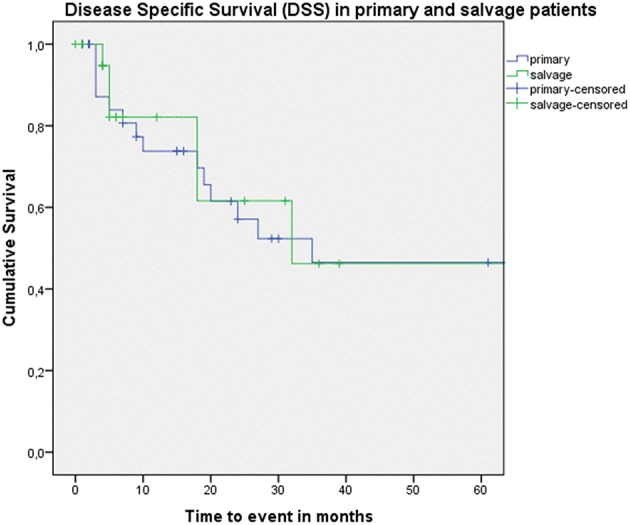
Kaplan–Meier curve illustrating disease-specific survival (DSS) in patients treated with up-front or primary surgery (blue) and salvage surgery (green). No difference in DSS between both groups is observed (log-rank test, *p* = 0.671).

**Figure 3:**
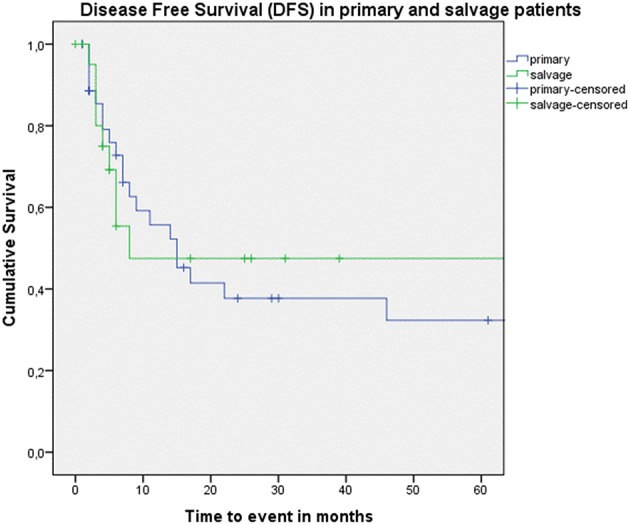
Kaplan–Meier curve illustrating disease-free survival (DFS) in patients treated with up-front or primary surgery (blue) and salvage surgery (green). No difference in DFS between both groups is observed (log-rank test, *p* = 0.835).

**Figure 4:**
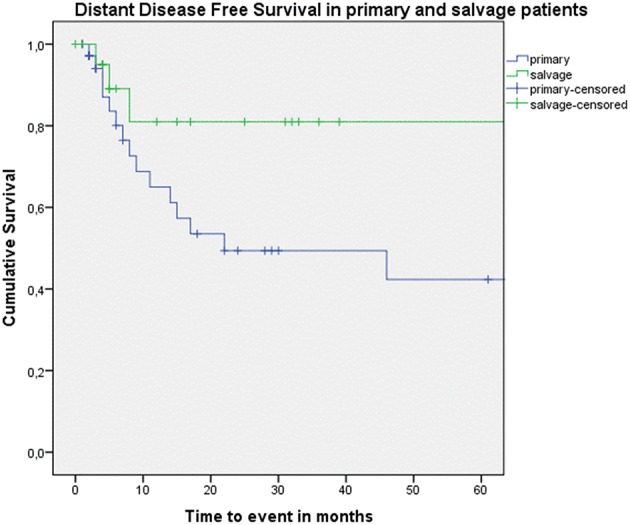
Kaplan–Meier curve illustrating distant disease-free survival (DFS) in patients treated with up-front or primary surgery (blue) and salvage surgery (green). A trend toward better distant DFS in the salvage group is observed, although this difference is not statistically significant (log-rank test, *p* = 0.078).

**Figure 5:**
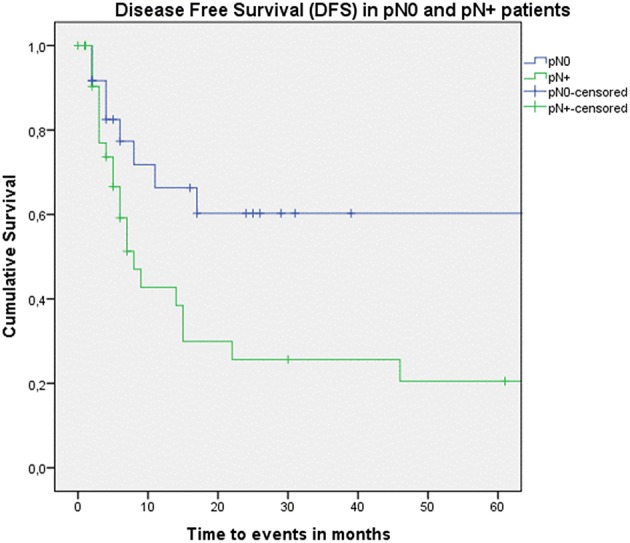
Kaplan–Meier curve illustrating disease-free survival (DFS) in pN0 patients (blue) vs. pN+ patients (green). A statistically significant better DFS in the pN0 group is observed (log-rank test, *p* = 0.015).

**Figure 6:**
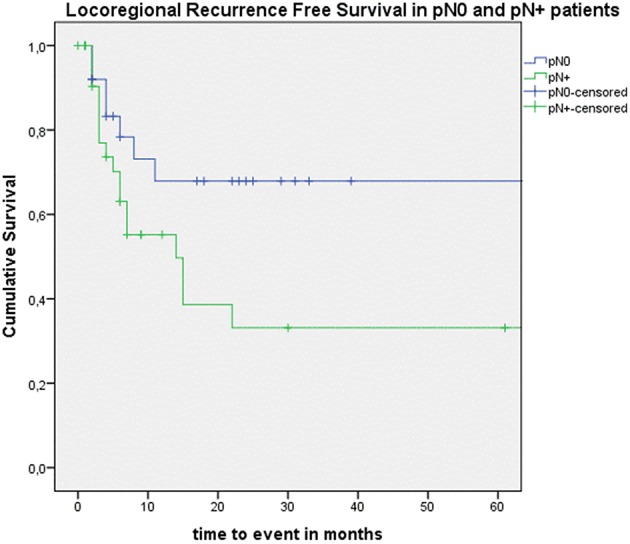
Kaplan–Meier curve illustrating locoregional recurrence-free survival (RFS) in pN0 patients (blue) vs. pN+ patients (green). A statistically significant better locoregional RFS in the pN0 group is observed (log-rank test, *p* = 0.049).

**Figure 7:**
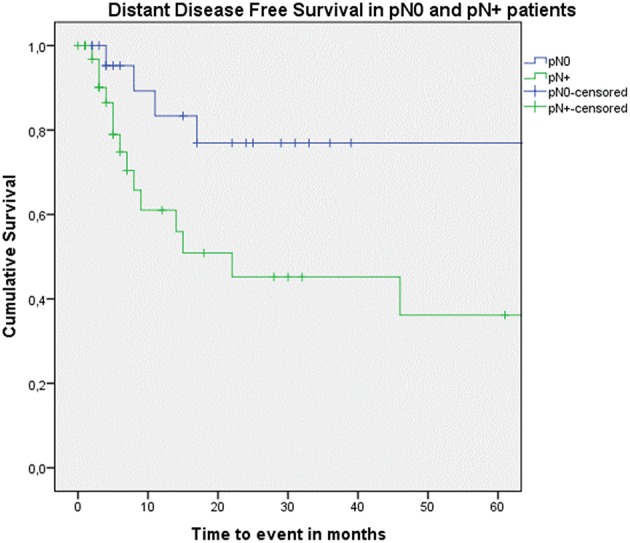
Kaplan–Meier curve illustrating distant disease-free survival (DFS) in pN0 patients (blue) vs. pN+ patients (green). A statistically significant better distant DFS in the pN0 group is observed (log-rank test, *p* = 0.015).

Looking at Kaplan–Meier survival estimates for the total population, estimated 2-year OS was 39.5% (SE = 6.4%), 2-year DSS was 58.5% (SE = 8.0%), and 2-year DFS was 40.3% (SE = 7.4%). Upon univariate analysis (log rank testing), no differences were observed in 2-year and 5-year OS (Figure 1), DSS (Figure 2), DFS (Figure 3) and locoregional RFS between the primary surgery and salvage surgery groups. However, a trend toward better distant DFS in the salvage group was observed, but this difference did not reach the significance level (log-rank test, *p* = 0.078) (Figure 4). 2-year and 5-year survival estimates (Kaplan Meier) in the total population, the primary surgery group and the salvage surgery group are summarized in Table 3. Upon univariate analysis, oncologic outcomes in pN0 patients proved favorable when compared with pN+ patient with a significant better DFS (*p* = 0.015) (Figure 5), locoregional RFS (*p* = 0.049) (Figure 6) and distant DFS (*p* = 0.015) (Figure 7) in the former patient group. Besides, near significant trends toward better OS and DSS were observed in the pN0 group. Table 4 depicts *p*-values after comparison of different oncologic outcome parameters between different subgroups using log-rank testing.

**Table 3:** Table depicting 2 and 5-year survival estimates (Kaplan Meier) in the total population, the primary surgery group and the salvage surgery group.

	**2-years**	**5-years**
OS total population	39.5% (SE = 6.4%)	21.1% (SE = 5.5%)
OS primary	45.2% (SE = 8.3%)	24.1% (SE = 7.3%)
OS salvage	30.4% (SE = 9.6%)	15.7% (SE = 7.9%)
DSS total population	58.5% (SE = 8.0%)	46.6% (SE = 8.9%)
DSS primary	57.1% (SE = 9.6%)	46.5% (SE = 10.4%)
DSS salvage	61.6% (SE = 14.4%)	46.2% (SE = 17.2%)
DFS total population	40.3% (SE = 7.4%)	35.2% (SE = 8.0%)
DFS primary	37.7% (SE = 9.0%)	32.3% (SE = 9.2%)
DFS salvage	47.5% (SE = 12.7%)	47.5% (SE = 12.7%)
Locoregional RFS total population	49.5% (SE = 7.6%)	49.5% (SE = 7.6%)
Locoregional RFS primary	46.7% (SE = 9.4%)	46.7% (SE = 9.4%)
Locoregional RFS salvage	57.3% (SE = 11.8%)	57.3% (SE = 11.8%)
Distant DFS total population	59.1% (SE = 7.9%)	51.7% (SE = 9.8%)
Distant DFS primary	49.4% (SE = 9.7%)	42.3% (SE = 10.6%)
Distant DFS salvage	81.0% (SE = 10.2%)	81.0% (SE = 10.2%)

*DFS, disease free survival; DSS, disease specific survival; OS, overall survival; RFS, recurrence free survival; SE, standard error*.

**Table 4:** Table depicting *p*-values after comparison of OS, DSS, DFS, locoregional RFS and distant DFS between different subgroups using log-rank test.

**Univariate analysis (log-rank)**	**OS**	**DSS**	**DFS**	**Locoregional RFS**	**Distant DFS**
Primary or salvage	*p* = 0.592	*p* = 0.671	*p* = 0.835	*p* = 0.808	*p* = 0.078
pN0 vs. pN+	*p* = 0.097	*p* = 0.058	***p*** **=** **0.015**	***p*** **=** **0.049**	***p*** **=** **0.015**

*DFS, disease free survival; DSS, disease specific survival; OS, overall survival; RFS, recurrence free survival. Bold p values indicate statistical significant differences on log-rank analysis (p < 0.05)*.

## Discussion

Last decades, cancers of the hypopharynx are, like cancers of the larynx, increasingly being treated with non-surgical larynx-preserving treatment modalities (radiotherapy with or without chemotherapy), obviously leading to a decrease in the rate of laryngopharyngectomies (11). Besides, early stage hypopharyngeal tumors, although relatively rare, are increasingly being treated with larynx sparing, minimal invasive surgery such as transoral laser microsurgery (TLM) or transoral robotic surgery (TORS) (12). However, laryngopharyngectomy is still a valuable treatment option for resectable failures of primary organ preservation protocols or in very advanced primary situations, in which a non-surgical organ preserving treatment regimen is unlikely to yield functional organ preservation. Of interest, a recent retrospective analysis of almost 4,000 patients with hypopharyngeal cancer (all stages) who were identified in the SEER (Surveillance, Epidemiology, and End Results) database, revealed an overall survival benefit for patients treated with combined surgery and radiotherapy when compared to other treatment modalities (11). In this analysis, 5-year overall survival of patients who underwent upfront surgery with adjuvant radiotherapy was 34.5% compared to 22.6% for patients who received upfront radiotherapy (with or without chemotherapy) (*p* < 0.001) (11). This survival benefit for the primarily surgically treated group could not be confirmed in a later retrospective comparative study comparing oncologic outcomes between 34 patients treated with upfront CRT vs. 57 patients treated with primary surgery followed by adjuvant RT or CRT. All patients had stage III-IV hypopharyngeal SCC and no differences between both groups were observed in 5-year local control, DFS and OS, while the functional larynx-preservation rate was higher in the CRT group (88.2 vs. 29.8%) (13). In our upfront surgery patient cohort, estimated 2-year and 5-year OS rates were 45.2% (SE = 8.3%) and 24.1% (SE = 7.3%) respectively. However, comparison with the aforementioned OS rates is difficult, mainly due to the fact that our group almost exclusively consists of advanced stage (stage III-IV) disease (97.3% of patients). On the other hand, our 5-year overall survival of the total population (21.1%) is comparable to overall survival rates reported in large surgical series including both primary and salvage patients: 24% in the series by Triboulet et al. and 24.5% in the series by Wei et al. (14, 15). Quite surprisingly in our patient series is that, upon comparing primary and salvage surgery subgroups, no differences were observed in 2-year and 5-year OS, DSS, DFS and locoregional RFS between both, intrinsically completely different, subgroups. A trend toward better distant DFS in the salvage group was observed, but this difference did not reach the significance level (log-rank test, *p* = 0.078). This can possibly be attributed to a selection of recurrent tumors without distant metastases in the salvage group. After all, of the patients who recur after primary (chemo) radiation, those with synchronous distant disease are considered no candidates for surgical salvage. In our overall patient series, pN+ status proved a statistically significant negative prognostic factor for DFS (*p* = 0.015), locoregional RFS (*p* = 0.049) and distant DFS (*p* = 0.015). In the aforementioned SEER data-analysis, N status (N2 or N3 relative to N0), together with age, race and treatment modality were significantly associated with worse OS upon multivariate analysis (11). This was also previously found by Saito et al, who identified presence of more than 3 metastatic lymph nodes, non-pyriform sinus locations and, in addition, presence of vascular invasion, as strong and significant negative prognostic factors in multivariate analysis of advanced hypopharyngeal carcinoma patients treated with total laryngopharyngectomy (16). As prognosis of hypopharyngeal cancer patients in need of total laryngopharyngectomy is poor, minimizing per- and postoperative morbidity, while achieving adequate functional results for swallowing and voice restoration is of the utmost importance. Nowadays, several reconstructive options exist for reconstructing a total laryngopharyngectomy defect, including gastric transposition or gastric pull-up, which is especially reserved for tumors with extension into the cervical esophagus, and free tissue transfer. Free flap reconstructive options (fasciocutaneous flaps such as tubed anterolateral thigh (ALT) or enteric flaps such as free jejunum) are in our center mainly used for reconstruction of a circular laryngopharyngectomy defect without extension into the cervical esophagus below the level of the cricopharyngeal muscle (primary cases) or in salvage cases in which the cricopharyngeal muscle is not invaded up to its lower border. The major disadvantage of gastric transposition is the relatively high rate of morbidity and mortality associated with this procedure involving three surgical fields (17). In our series, in hospital mortality was 8.3% and early head and neck complications were recorded in 15% of patients with 35% of patients additionally experiencing early thoraco-abdominal complications. This is comparable to published data by Wei et al. who reported a significant reduction in postoperative mortality (31–9%) and serious morbidity such as anastomotic leakage and bleeding (20–10%) in their series of 317 patients treated with gastric transposition over a 30-year period. However, overall minor complication rate remained at about 49% (15). In another large series including 127 patients who underwent laryngopharyngoesophagectomy with gastric transposition, Triboulet et al. reported an in-hospital mortality rate of 4.8% and a complication rate of 33.1%, with anastomotic leakage and pulmonary complications being the most common (14). As most recent surgical series on advanced hypopharyngeal cancer include cases with and without extension into the cervical esophagus, different reconstructive techniques are used (including free tissue transfer and gastric transposition) and comparative data regarding morbidity and postoperative functionality between different reconstructive techniques have been published. It is generally believed that gastric transposition entails a more significant postoperative morbidity when compared to free tissue transfer reconstructions, but data in the literature are contradictory. In a retrospective comparative study including 68 patients with a total laryngopharyngectomy defect, gastric pull-up reconstruction independently predicted for increased wound complications (*p* = 0.014 with odds ratio (OR) of 8.26), PCF (*p* = 0.012, OR = 7.63) and trended toward predicting early total morbidity (*p* = 0.085, OR = 4.93) on multivariate analysis (18). However, the authors reported a pharyngocutaneous fistula rate of 48% in gastric transposition patients as opposed to a 27% rate in patients who underwent free flap reconstruction of partial and total laryngopharyngectomy defects, compared to an 8.3% rate in our population, 15.7% in the large series by Triboulet et al. and 9–31% in the series by Wei et al. (14, 15). To the contrary, other studies report higher rates of PCF after free tissue transfer reconstruction, even up to 71.4% for tubed radial forearm flaps in the salvage setting (19). Triboulet et al. and Iseli et al. reported more complications, including flap necrosis, and fistulas, leading to delayed feeding, in patients who underwent free jejunal transfer when compared to gastric transposition (14, 17). Concerning postoperative functional outcomes, complete oral intake was achieved in 82.7% of patients. Despite the idea that the reconstruction with a capacious stomach pull-up could lead to an ideal mucosally lined conduit, successful swallowing after gastric transposition ranges from 71 to 100% (17, 20). With regards to speech rehabilitation, we try to rehabilitate all our laryngopharyngoesophagectomy patients with electrolarynx speech. After all, electrolarynx speech remains an important, frequently used and very viable communication option for patients who undergo total laryngectomy, although considerable differences in electrolarynx performance among subjects exist (21). In patients who fail rehabilitation with electrolarynx or are unhappy with quality of the resulting speech, secondary puncture in the septum between the transposed stomach and the trachea with introduction of an indwelling voice prosthesis (Provox®) is offered. Of these patients with a voice prosthesis, 68.0% achieved a functional speech rehabilitation. Little is known about puncture voice restoration after gastric transposition. In a series by Keereweer et al. reporting on oncologic and functional outcomes after total laryngopharyngectomy including 51 jejunum interpositions and 19 gastric pull-up procedures, successful speech rehabilitation is reported in 95% of patients, of which 52% communicated by the use of electrolarynx and 43% by the use of a voice prosthesis. However, no details about speech rehabilitation in the gastric pull-up subgroup are reported (22). The same is true for the series by Clark et al, who reports voice restoration using puncture and valve insertion in 44% of the total patient population, including gastric transposition cases as well as free tissue transfer cases (18). In a small cohort reported by Iseli et al., none of 7 gastric transposition patients received a voice prosthesis, but all succeeded in speech rehabilitation, either by “esophageal” speech or by electrolarynx speech (17). When focusing on speech rehabilitation results after laryngopharyngectomy with free flap reconstruction, a recent systematic review reported an overall rehabilitation rate by tracheo-esophageal puncture speech of 36% (23). Compared to these data, postoperative voice rehabilitation after gastric transposition seems non-inferior, especially when voice prosthesis placement is considered in these patients who fail electrolarynx speech. However, potential complications need to be taken into account.

Evidently, our study has limitations. As a retrospective study, inherent selection bias cannot be excluded. Moreover, because of the relatively small study population and small subgroups (salvage vs. primary surgery group), multivariate analysis was not possible. Additionally, during data retrieval, we observed high rates of lacking data concerning functional rehabilitation (swallowing and speech). Another drawback of our study is the lack of objective postoperative voice assessments.

## Conclusion

In a time of larynx-preserving regimens to treat advanced hypopharyngeal SCC, laryngopharyngoesophagectomy with gastric pull-up reconstruction is still a valuable treatment option for resectable failures of primary organ preservation protocols, or in very advanced primary situations, in which an organ preserving treatment regimen is unlikely to yield functional organ preservation. It combines acceptable oncologic and functional outcomes in an prognostic unfavorable patient group. However, as this is a very invasive procedure with significant perioperative mortality and overall morbidity, tumor board discussion, and patient selection is of the utmost importance in order to achieve an acceptable functional and oncological outcome.

## Synopsis

Total laryngopharyngoesophagectomy with gastric pull-up reconstruction for advanced stage hypopharyngeal SCC combines relatively good oncologic and functional outcomes in a prognostically unfavorable patient group, both in the primary and the salvage setting.

## Data Availability

The datasets generated for this study are available on request to the corresponding author.

## Ethics Statement

This study was approved by and carried out in accordance with the recommendations of the Institutional Review Board (University Hospital Leuven Committee for Medical Ethics). Informed consent was waived given the retrospective nature of this study.

## Author's Note

The abstract of this paper was presented by JM as an oral communication during the IFOS world ENT conference organized in Paris, France (24–28th of June 2017).

## Author Contributions

JM contributed to study set-up, data quality control, data analysis (statistics), drafting manuscript, and review of manuscript. FC and EB contributed to data collection, data analysis (statistics), and drafting manuscript. PN, HV, VV, and PD contributed to drafting manuscript and review of manuscript. WC contributed to study set-up, drafting manuscript, and review of manuscript.

### Conflict of Interest Statement

The authors declare that the research was conducted in the absence of any commercial or financial relationships that could be construed as a potential conflict of interest.
